# Totally Endoscopic Minimally Invasive Aortic Valve Replacement Under Near Normothermia in a Patient With Cryoglobulinemia

**DOI:** 10.7759/cureus.111905

**Published:** 2026-07-01

**Authors:** Masaaki Kobayashi, Satoshi Arimura, Toshiaki Ito

**Affiliations:** 1 Department of Cardiovascular Surgery, Shonan Kamakura General Hospital, Kanagawa, JPN

**Keywords:** cryoglobulinemia vasculitis, minimal invasive cardiac surgery, on-pump cardiac surgery, surgical aortic valve, targeted normothermia

## Abstract

Cardiac surgery in patients with cryoglobulinemia remains challenging because perioperative hypothermia may increase the risk of cryoglobulin-related complications. We report the case of a 49-year-old man with severe bicuspid aortic valve stenosis and preoperatively confirmed cryoglobulinemia who underwent totally endoscopic minimally invasive aortic valve replacement without preoperative plasmapheresis. The patient had no clinical manifestations of cryoglobulinemic vasculitis, and extensive hematologic and immunologic evaluation demonstrated no monoclonal gammopathy, a normal serum free light chain ratio, and negative viral and autoimmune markers. Because the patient was considered low risk for cryoglobulin-related complications, surgery was successfully performed using normothermic cardiopulmonary bypass with intermittent tepid blood cardioplegia, while maintaining a lowest core temperature of 34.8 °C. The postoperative course was uneventful, with no evidence of cryoglobulin-related complications. This case suggests that, in carefully selected low-risk patients without active vasculitic manifestations, minimally invasive cardiac surgery under strict normothermic management may be feasible without routine preoperative plasmapheresis.

## Introduction

Cryoglobulinemia is a rare immunologic disorder defined by the presence of circulating immunoglobulins that reversibly precipitate at temperatures below 37 °C and dissolve upon rewarming [1,2]. These cryoglobulins may form immune complexes that deposit within small and medium-sized vessels, leading to endothelial injury, vasculitis, thrombosis, and multiorgan dysfunction [1,2]. Although cardiac involvement is relatively uncommon, cardiac surgery represents a particularly challenging setting because systemic hypothermia and cold cardioplegia are commonly used during cardiopulmonary bypass, potentially promoting cryoglobulin precipitation and microvascular complications [1-4].

Several perioperative strategies have been proposed to reduce the risk of cryoglobulin precipitation, including preoperative plasmapheresis, immunosuppressive therapy, and tailored temperature management [1,4]. However, evidence supporting standardized approaches is limited, and no consensus exists regarding optimal risk stratification or prophylactic interventions, particularly in asymptomatic patients [1,4]. We report a case of totally endoscopic minimally invasive aortic valve replacement performed under near-normothermic cardiopulmonary bypass with tepid blood cardioplegia, without preoperative plasmapheresis, in a patient with known cryoglobulinemia.

## Case presentation

A 49-year-old man was referred to our institution for surgical treatment of severe aortic stenosis associated with a bicuspid aortic valve. His medical history was significant for diabetes mellitus, diabetic nephropathy, and nephrotic syndrome. Cryoglobulinemia had been diagnosed during the evaluation of renal dysfunction based on a positive qualitative serum cryoglobulin test, although the subtype had not been specified. Preoperative evaluation revealed no clinical features suggestive of cryoglobulinemic vasculitis, including palpable purpura, arthralgia, peripheral neuropathy, or systemic vasculitic manifestations. Hematological assessment showed no evidence of an underlying hematologic malignancy: serum and urine immunofixation electrophoresis did not detect monoclonal protein, and the serum free light chain ratio was within the normal range. Serological testing was negative for human immunodeficiency virus, hepatitis B virus, and hepatitis C virus. Rheumatoid factor was also negative.

**Figure 1:**
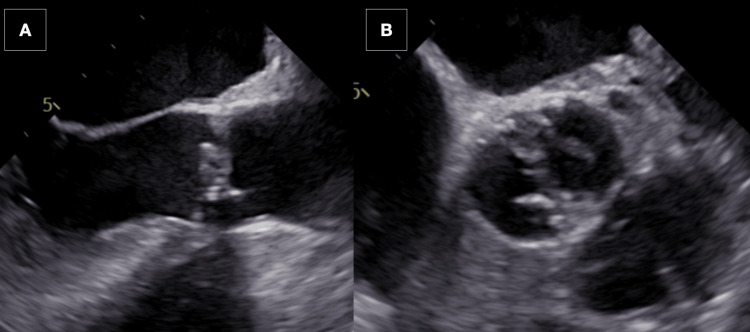
Preoperative transesophageal echography (A) Long-axis view of the aortic valve demonstrating restricted leaflet opening. (B) Short-axis view showing a bicuspid aortic valve with limited systolic excursion.

Preoperative echocardiography demonstrated severe bicuspid aortic valve stenosis, with a peak transvalvular velocity of 4.0 m/s, a mean pressure gradient of 43 mmHg, and an effective orifice area index of 0.87 cm²/m² (Figure 1). No significant aortic regurgitation was observed. Left ventricular systolic function was mildly reduced, with a left ventricular ejection fraction of 49%. Given the diagnosis of cryoglobulinemia and the potential risk of cryoglobulin precipitation during hypothermic exposure on cardiopulmonary bypass, perioperative management was carefully discussed in a multidisciplinary conference involving cardiac surgeons, anesthesiologists, perfusionists, and hematologists. Particular attention was directed toward minimizing perioperative temperature fluctuations and avoiding cold exposure throughout the surgical procedure. In the absence of active cryoglobulinemic vasculitis or other high-risk clinical or hematologic features, the multidisciplinary team determined that preoperative plasmapheresis was not mandatory, and the patient was scheduled for totally endoscopic minimally invasive aortic valve replacement under near-normothermic management.

**Figure 2:**
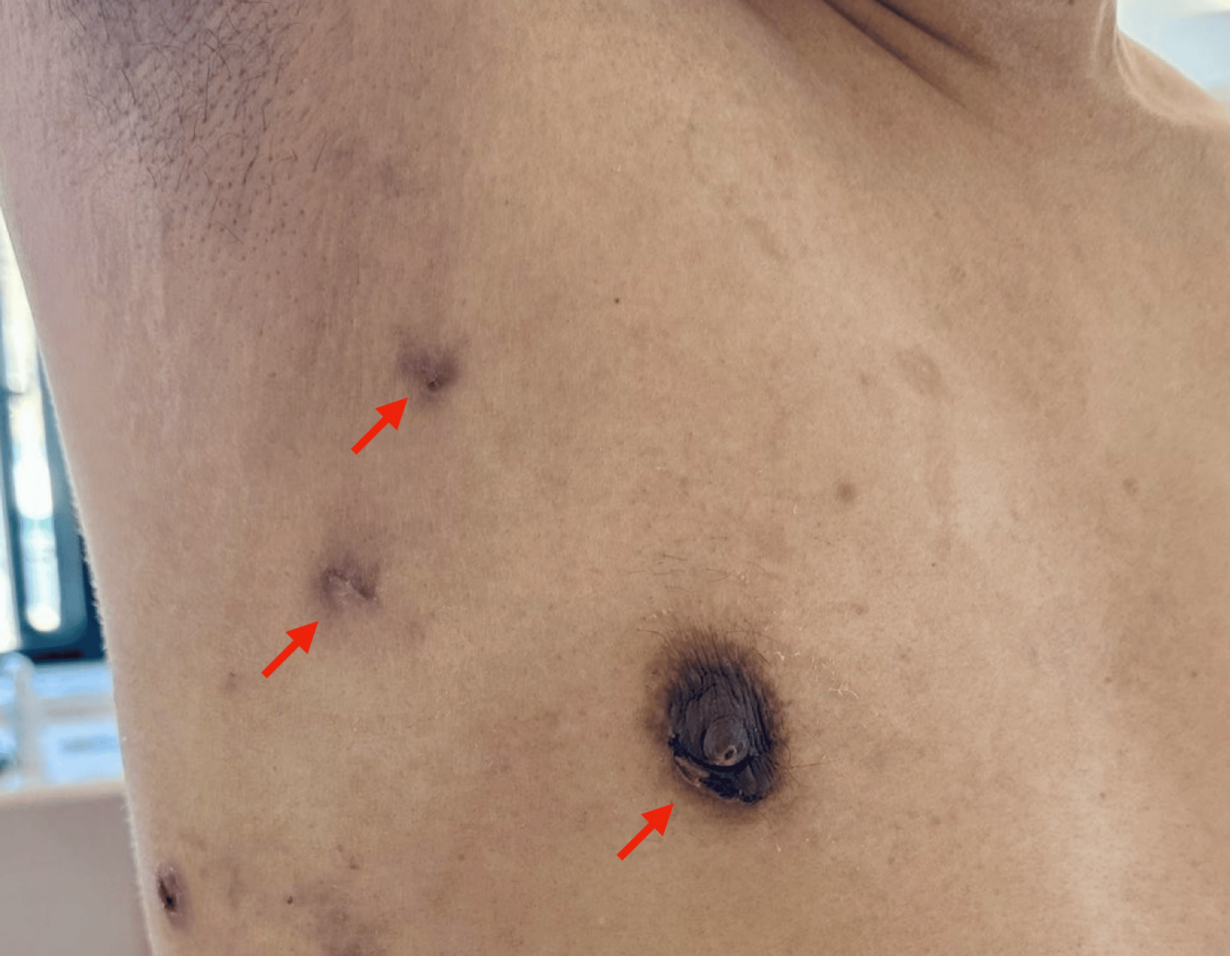
Postoperative incision appearance after totally endoscopic minimally invasive aortic valve replacement. The red arrows indicate the skin incision.

The patient underwent totally endoscopic minimally invasive aortic valve replacement through a right mini-thoracotomy using a periareolar approach, with additional small port incisions at the second and third intercostal spaces (Figure 2). Cardiopulmonary bypass was established using femoro-femoral cannulation. Systemic temperature was meticulously controlled and maintained within the near-normothermic range, with careful avoidance of hypothermia. The lowest recorded bladder temperature during cardiopulmonary bypass was 34.8 °C.

**Figure 3:**
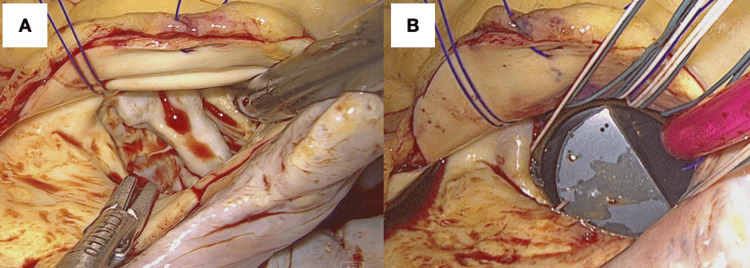
Intraoperative view (A) Heavily calcified bicuspid aortic valve. (B) Intraoperative view after the aortic valve replacement with a mechanical valve.

Myocardial protection was achieved using tepid blood cardioplegia at 32 °C, administered intermittently at 20-minute intervals. Cold cardioplegia was deliberately avoided to minimize the risk of cryoglobulin precipitation. After excision of the native bicuspid aortic valve, a 23-mm mechanical prosthetic valve (St. Jude Medical Regent; Abbott, Chicago, IL, USA) was implanted in the supra-annular position (Figure 3).

The total operative time was 182 minutes. The cardiopulmonary bypass time was 126 minutes, and the aortic cross-clamp time was 89 minutes. The patient was weaned from cardiopulmonary bypass without difficulty. No intraoperative findings suggestive of cryoglobulin precipitation, hemolysis, or microvascular thrombosis were observed.

The postoperative course was uneventful. Renal function remained stable, and no thrombotic or ischemic complications occurred. Histopathological examination of the myocardial biopsy specimens did not demonstrate definitive evidence of cardiac amyloidosis. The patient was discharged in stable condition on postoperative day 10.

## Discussion

Cryoglobulinemia presents a unique challenge in cardiac surgery because hypothermia can induce immunoglobulin precipitation, leading to microvascular obstruction and end-organ ischemia [1,2]. During cardiopulmonary bypass, even mild reductions in temperature may promote cryoglobulin aggregation, and several reports have described severe perioperative complications associated with hypothermic management [3,4].

Previous reports have emphasized that perioperative management should be individualized according to the clinical severity of cryoglobulinemia, the cryoglobulin burden, and the planned cardiac procedure. A recent single-center experience and literature review highlighted various case-specific modifications, including preoperative or postoperative plasmapheresis, immunosuppressive therapy when indicated, normothermic cardiopulmonary bypass, warm blood cardioplegia, avoidance of systemic hypothermia, controlled rewarming, and multidisciplinary perioperative planning [1,4]. However, there is no consensus regarding the optimal perioperative strategy, and the predictive value of laboratory assessments such as cryocrit level, thermal amplitude testing, or cryoglobulin subtype for perioperative risk stratification remains unclear [1,5].

In the present case, quantitative assessment of cryoglobulins was not available at our institution, representing an important limitation. Nevertheless, extensive clinical and laboratory evaluation did not identify features suggestive of high-risk cryoglobulinemia, such as active cryoglobulinemic vasculitis, monoclonal gammopathy, abnormal free light chain ratios, or infection-related etiologies [2]. Furthermore, preoperative plasmapheresis and immunosuppressive therapy are not without risk and may be associated with procedural complications, infection, hemodynamic instability, or delays in definitive surgical treatment [4].

At our institution, hypothermic cardiopulmonary bypass with cooling to 32 °C is generally employed during minimally invasive cardiac surgery to reduce the risk of re-expansion pulmonary edema. Considering the absence of high-risk clinical features and the lack of evidence supporting routine preoperative intervention in low-risk patients, we concluded that maintaining near-normothermia during cardiopulmonary bypass represented the most pragmatic strategy for minimizing perioperative risk.

Although cryoglobulin precipitation was not observed in the present case, prompt rewarming and avoidance of further cooling would be the initial management if precipitation were suspected intraoperatively. Panagiotopoulos et al. reported successful resolution of unexpected intraoperative cold-reactive protein precipitation during cardiac surgery following immediate rewarming with warm saline infusion and restoration of normothermia [6].

Minimally invasive aortic valve replacement is associated with slightly longer cardiopulmonary bypass and aortic cross-clamp times than conventional sternotomy. A recent meta-analysis reported mean increases of 7.8 minutes for cardiopulmonary bypass and 6.0 minutes for aortic cross-clamp time [7]. These modest increases are offset by reduced blood loss, lower transfusion requirements, and faster postoperative recovery, without compromising early clinical outcomes. In the present case, our primary objective was not to minimize cardiopulmonary bypass or aortic cross-clamp time, but to avoid hypothermic exposure, the principal trigger for cryoglobulin precipitation. Therefore, we considered totally endoscopic minimally invasive aortic valve replacement performed under near-normothermic conditions to be an appropriate surgical strategy for this patient.

In our institution, cold blood cardioplegia at approximately 15 °C is routinely used for minimally invasive aortic valve replacement. In this case, we instead selected tepid blood cardioplegia at 32 °C to minimize hypothermic exposure while maintaining adequate myocardial protection. Although normothermic cardioplegia may also be a reasonable option, tepid cardioplegia was considered more practical because it requires less frequent redosing.

This report is limited by its single-case nature, and the safety and efficacy of this approach cannot be generalized. Nevertheless, this experience suggests that near-normothermic cardiopulmonary bypass with tepid myocardial protection may represent a feasible management option in selected patients with cryoglobulinemia. We present this case to contribute to the limited literature and to support individualized perioperative decision-making in similar clinical scenarios.

## Conclusions

This case demonstrates the feasibility of totally endoscopic minimally invasive aortic valve replacement in a selected patient with cryoglobulinemia when perioperative temperature is carefully controlled. Because the patient had no active vasculitis, monoclonal gammopathy, or other high-risk features, the procedure was performed without preoperative plasmapheresis. Instead, management focused on minimizing cold exposure through near-normothermic cardiopulmonary bypass and 32°C blood cardioplegia. Although the findings from a single case cannot be generalized, careful preoperative risk assessment, multidisciplinary planning, and strict attention to temperature management may be important when cardiac surgery is required in patients with cryoglobulinemia.
